# Mechanical and Microstructural Properties of Alkali-Activated Biomass Fly Ash and Diatomite Blends

**DOI:** 10.3390/ma18163807

**Published:** 2025-08-13

**Authors:** Darius Žurinskas, Danutė Vaičiukynienė

**Affiliations:** Faculty of Architecture and Civil Engineering, Kaunas University of Technology, Studentų St. 48, 51367 Kaunas, Lithuania; danute.vaiciukyniene@ktu.lt

**Keywords:** alkali-activated binder, blends of biomass fly ash and calcined diatomite, aluminosilicate precursor, microstructure, compressive strength, binding phases

## Abstract

Biomass is one of the most important sources of renewable energy, generating large amounts of ash. This increases the amount of waste, landfill, and air pollution. This work focuses on the sustainable disposal of this ash by producing an innovative binder. The mechanical and microstructural properties of alkali-activated biomass fly ash (BFA) and diatomite (DT) mixtures are currently insufficiently studied. New scientific knowledge of these properties is needed. This study presents the possibility of using BFA and diatomite as aluminosilicate precursors for the production of an alkaline-activated binder. It was found that the reactivity of BFA is relatively low. Based on XRD analysis, the mineral composition of BFA is dominated by quartz and calcite, both of which are non-reactive minerals. Therefore, mixtures with DT were created as precursors. According to Rietveld analysis data, an amorphous part was found in both precursor materials, BFA and DT. Comparing the chemical composition of BFA and DT using XRF and Rietveld analysis data, it was found that the amorphous part of BFA consists of CaO, while the amorphous part of DT consists of SiO_2_. Thus, the combination of these precursors should complement each other during the geopolymerisation process. After 28 days of curing, the strength of the binders was dependent on the amount of DT, and the highest strength values, such as 16.4 MPa and 15.3 MPa, were obtained when DT contents were 10% and 30%, respectively. After geopolymerisation, XRD analysis showed that calcium silicate hydrate, hydrotalcite, and calcium aluminium silicate hydrate (zeolite A type) were formed. SEM analysis confirmed the XRD results and showed that DT additives (10% and 30% by weight) improved the microstructure of alkali-activated BFA, which is closely related to compressive strength values. The proposed binder will be useful in the preparation of concrete, which could be used for artificial aggregates or small architectural elements.

## 1. Introduction

In Lithuania, most biomass ash ends up in landfills. This is inappropriate storage of biomass ash, as it increases the size of landfills and underutilises secondary raw materials such as biomass ash. The reactivity of biomass wood ash is relatively low, so the development of multi-component aluminosilicate blends is recommended for better mechanical properties. Due to the low reactivity of biomass wood ash, the compressive strength of alkali-activated binders made exclusively from this ash as a precursor ranges from 8.5 to 22.0 MPa. The chemical and mineral composition of biomass wood ash is closely related to its strength properties, as stated by Du et al. [1]. According to Martínez-García et al. [2], the use of biomass wood ash in the production of geopolymers is limited, and the particle size, shape, and preparation method of the ash, which are closely related to the chemical and mineral composition, have a significant impact on the basic properties of the geopolymer samples. Liu et al. [3] gave an overview of the use of silica-rich biomass ash in geopolymer preparation. They stated that these biomass ashes can be used as a precursor and as an alkali activator, as the ashes can be used to produce a sodium silicate solution. A geopolymer based on biomass and fly ash has been studied by Songpiriyakij et al. [4]. The study demonstrated that the reactivity of the initial materials and the quality of the matrix exerted a significant influence on the enhancement of the compressive strength of the geopolymer samples. In the study by Alonso et al. [5], the olive biomass fly and bottom ash were used to prepare an alkaline activator, which was used in the preparation of alkaline-activated materials. Using this alternative alkaline activator in blast furnace slag systems, samples with compressive strengths up to 33 MPa were formed. In the study [6], the alkali-activated material was made from high-carbon biomass ash as a precursor, and a mixture of Na_2_CO_3_ and soluble glass was used as an alkaline activator. The maximal compressive strength reached 3.5 MPa after 28 days of hydration.

Compared to other supplementary cementitious materials (SCMs), such as red mud, BFA has a mineral composition dominated by crystalline compounds with low reactivity. In this case, BFA and red mud are similar aluminosilicate precursors, and using them as separate aluminosilicate precursors does not allow for high strengths to be achieved during geopolymerisation. However, as Guo et al. [7] pointed out, red mud contains a significantly higher amount of Al_2_O_3_ and, under optimum conditions, can produce more geopolymerisation products with higher mechanical properties than BFA. In another study, Li et al. [8] proposed the use of a complex alkaline activator: NaOH, Na_2_O∙nSiO_2_, and Na_2_CO_3_ to improve the geopolymerisation of red mud. When comparing fly ash (from coal combustion) and slag with BFA, these aluminosilicates are well-known high-reactivity pozzolanic materials, which are often used in combination with low-reactivity precursors such as red mud or biomass ash.

The most common alkali-activated aluminosilicate binders are produced not only from biomass wood ash but also from mixed precursors using other aluminosilicate materials. Silvestro et al. [9] made geopolymer from metakaolin and biomass wood ash blends where 10 and 20 wt% of metakaolin was substituted with biomass wood ash. The results showed that the strength values of the samples made from metakaolin and wood biomass ash were similar to those of the reference samples without metakaolin. A dry activator made from pretreated wood biomass ash with NaOH was mixed with diatomite, as an aluminosilicate precursor, to produce dry cement, according to Hassan et al. [10]. This ash was treated with mixtures of sodium NaOH and CaCO_3_ and then mixed with diatomite. The strength properties were closely linked to the formation of calcium silicate hydrate and calcium aluminosilicate hydrate, which are responsible for developments in strength. The maximal compressive strength was 48 MPa after 28 days. Bijeljić et al. [11] investigated alkali-activated blends of aluminosilicate precursors made from coal fly ash by replacing them with biomass wood ash. This substitution had a positive impact on the development of strength, especially at an early age of the samples, when up to 25% biomass wood ash was included in the systems. The glass powder, as an additional precursor material together with wood biomass ash, was used for the production of alkali-activated materials as reported by Silva et al. [12]. The compressive strength of alkali-activated wood biomass ash ranged from 2.85 to 3.69 MPa when sodium hydroxide was the alkali activator. The use of glass powder led to positive development of compressive strength, and in this case, the strength range was 7.20–19.62 MPa.

Diatomite is a natural material found all over the world. This material was formed from single-celled algae that vary in shape and size. Diatomite is very porous, and its main chemical compound is amorphous silica gel. Sometimes, as a natural material, it contains certain impurities, such as quartz, clay minerals, or calcite. The high amorphous silica content in diatomite makes it attractive for use as a precursor in the production of alkali-activated binders. İlkentapar et al. [13] produced geopolymer mortars from coal fly ash by substituting it with some amount of diatomite. The diatomite substitute had a positive effect on the development of the mechanical properties of geopolymer mortars. The optimal amount of substitute was 2% by weight, and the samples were cured at 60 °C for 48 h using 10% Na systems. Another study [14] found that substituting fly ash with diatomite improved the workability, unit weight, and strain capacity of geopolymer mortar. Özsoy et al. [15] found that geopolymer mortars in which diatomite replaced 1% and 2% of coal fly ash increased the compressive and flexural strength, but a higher diatomite content reduced the mortar strength. In this case, the addition of diatomite promotes the formation of amorphous (NASH gel) and crystalline (sillimanite) hydration products, which had a positive effect on the dense microstructure and strength values. Nykiel et al. [16] developed geopolymers from fly ash (coal) or metakaolin as an aluminium silicate precursor with 1–3% by weight of calcined diatomite as an additive. It was found that the addition of diatomite as a precursor had a negative effect on the strength properties of the samples. However, in cases where diatomite was used as an aggregate instead of sand (5% by weight), the strength increased by up to 24% (about 52 MPa) compared to samples in which only sand was used as an aggregate. In a similar study [17], calcined and non-calcined diatomite was added to the geopolymer mortar instead of sand. The best compressive strength was 34 MPa.

In most cases, scientific articles state that calcined diatomite improves the mechanical properties of alkaline-activated systems [18,19,20]. In study [18], dolomite waste was mixed with metakaolin and diatomite. Diatomite has a negligible effect on early-stage strength properties, but after 28 days of hydration, it has a significant positive effect on strength. This can be explained by the fact that amorphous compounds from diatomite dissolve slowly in an alkaline environment, and after a longer curing time (28 days), calcium and silica dissolve and form calcium aluminium/silicate hydrates, which strengthen the matrix. Ilkentapar et al. [21] substituted fly ash with diatomite in the preparation of geopolymer mortar. In this case, 1%, 2%, and 3% diatomite increased the strength values, which can be explained by the higher specific surface area of the diatomite. Bagci et al. [22] investigated a geopolymer based on metakaolin as a precursor, and calcined diatomite dissolved in a KOH solution was used as an alkali activator. The diatomite was calcined at 400 °C in aluminium foil, and finally, the diatomite powder was dissolved in an alkali solution. A similar alkaline activator was obtained by dissolving fumed silica in an alkaline solution. The self-reinforcement of the unreacted diatomite in the geopolymer matrix resulted in a significantly higher compressive strength (71 MPa) compared to the geopolymer produced without diatomite (54 MPa) after curing at room temperature. In study [23], foamed geopolymers were produced by using diatomite (5%, 10%, and 50% of the diatomite were substituted with fly ash) in the fly ash geopolymer. As a foaming agent a H_2_O_2_ solution was used, the precursor was activated with a 10 M NaOH solution. The thermal conductivity coefficient, density, and compressive strength were optimal by introducing 5% diatomite in the geopolymer system.

This work aims to examine the role of calcined diatomite on the main properties of biomass wood fly ash. The alumina silicate precursor was based on this ash by substituting with varying weight percentages of diatomite powder. The mixtures obtained were activated with a sodium hydroxide solution, then cured for one day at 60 °C and left at room temperature for 27 days.

## 2. Materials and Methods

### 2.1. Materials

Two source materials are used: biomass fly ash (BFA) and calcined diatomite (DT). BFA is produced by burning coniferous wood waste chips to produce hot water. The boiler plant uses fluidised bed technology, and the temperature of the quartz sand bed is maintained at around 800 °C. The boiler plant with this technology is located in Elektrėnai, Lithuania. Diatomite is obtained from a Lithuanian manufacturer, calcined at a temperature of 800 °C, and ground and sieved through a 0.63 mm sieve.

The chemical composition of BFA is characterised by a high content of CaO, which can be considered to be practically equal to the content of SiO_2_. The chemical composition also contains other oxides, such as K_2_O, Al_2_O_3_, MgO, and SO_3_, but the amount is significantly lower compared to CaO and SiO_2_. However, the heavy metals were detected in the chemical composition of BFA. Geopolymers are promising materials for immobilising heavy metals. The products of geopolymerisation are typically calcium silicate/aluminium hydrate and sodium silicate/aluminium hydrate, which are well-known heavy metal encapsulants [24,25,26,27].

The chemical composition of the DT is dominated by SiO_2_ with traces of Al_2_O_3_ and FeO. The detailed compositions were analysed by X-ray fluorescence (XRF) and are given in Table 1.

The mineral composition of BFA is dominated by quartz crystals. Some of the free lime reacted with CO_2_ to form calcium carbonate. Microcline and periclase were also found in the composition. A similar mineral composition for wood biomass ash was found by Silva et al. [12], where XRD patterns were dominated by quartz with calcium compounds such as calcite and portlandite.

The mineral composition of DT is completely dominated by quartz. Some muscovite and sanidine were present. It is possible that sanidine formed during the calcination process [28]. A similar mineral composition was found by Vasile et al. [29]. Some types of clay minerals (montmorillonite, illite, and kaolinite) and quartz with amorphous components (aluminosilicates, aluminium, and iron hydroxides) were identified in the studied DT.

**Figure 1:**
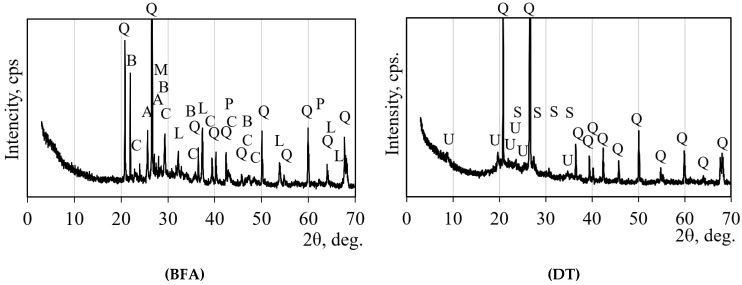
Mineral composition of biomass fly ash (BFA) and diatomite (DT) according to X-ray diffraction (XRD). Notes: Q is quartz, C is calcite, B is cristobalite, L is lime, M is microcline, P is periclase, A is α-quartz, U is Muscovite, S is sanidine.

The X-ray diffraction (XRD) diagrams of the mineral composition of BFA and DT with the abbreviations of the mineral peaks are shown in Figure 1. A full description of the mineral abbreviations is given in Table 2, and the percentages of the minerals and amorphous fraction obtained from the Rietveld analysis are also given in this table.

A comparison of the chemical compositions of BFA and DT by Rietveld analysis and XRF (Table 1) led to the conclusion that the amorphous part of BFA consists of CaO while the amorphous part of DT consists of SiO_2_.

**Figure 2:**
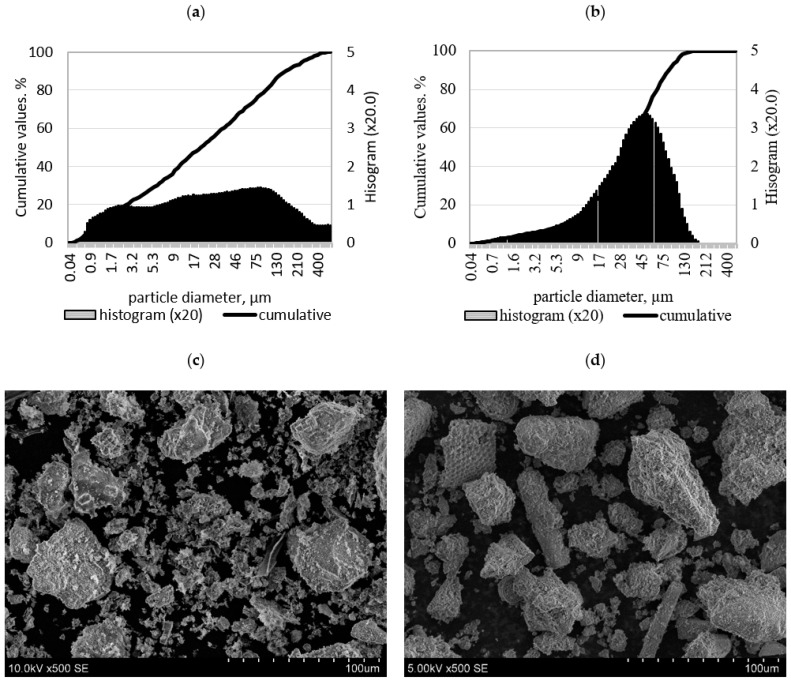
Granulometric composition of BFA (**a**) and DT (**b**) and microstructure of BFA (**c**) and DT (**d**).

The results of granulometric composition analysis showed that after milling, the BFA particle size ranged from 0.04 µm to more than 400 µm, without forming sharp peaks in the histogram (all particles were distributed evenly) (Figure 2a). A narrower range had DT, which was in the range of 0.04 µm and 130 µm with sharp peaks at about 45 µm (Figure 2b). Based on the mean diameter of the particle, DT had a lower value than BFA, which was 41.42 µm and 58.59 µm, respectively. However, BFA particles were almost twice as fine (a specific surface area of 3958 cm^2^/g) as DT particles (a specific surface area of 1959 cm^2^/g).

According to SEM BFA, it consists of particles with irregular shapes and with a wide range of visible aggregations (Figure 2c).

The morphology of the diatomite particles was quite variable. Porous and irregular sharp-edged and spongy particles were observed in the material, forming agglomerates (Figure 2d). A similar morphology of a fossil algae type of DT was detected by Bagci et al. [22].

Therefore, the combination of these two initial materials as precursors could be promising for the production of alkaline-activated binders.

### 2.2. Preparation and Test of the Samples

The binder samples were formulated in three main groups: 10%, 30%, and 50% DT added to BFA. The mixed bulk of each group is activated with a NaOH solution of constantly increasing concentration. The compositions of the samples are described in Table 3.

**Figure 3:**
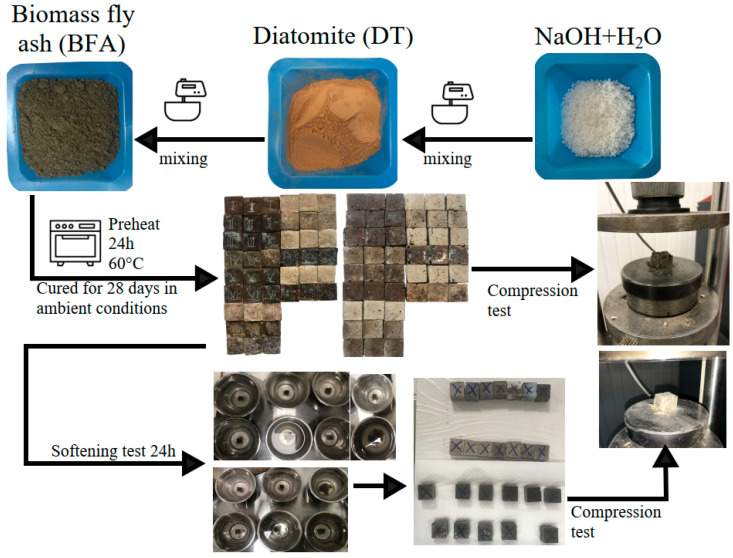
The sequence of sample preparation.

After that the pastes were poured into 20 × 20 × 20 mm moulds, and the moulds were placed on a vibrating plate until the extra air bubbles were removed. After that the samples are sealed in plastic bags and left to cure for 24 h at ambient conditions. After 24 h, the samples in the bags are placed in a heating oven for 24 h at 60 °C. After heating the samples again, the samples were cured for 7 and 28 days after moulding under ambient conditions. A graphical representation of the initial materials and the overall sequence of sample formation is given in Figure 3.

Finally, the samples were dried for at least 24 h at a temperature of 40 to 60 °C before being subjected to compression or softening tests.

### 2.3. Experimental Techniques

The elemental composition of the precursor’s materials was analysed by X-ray fluorescence spectrometry (XRF). The XRF analysis of the starting materials was carried out on a Bruker X-ray S8 Tiger WD using a rhodium (Rh) tube, with an anode voltage (Ua) of up to 60 kV and an electric current (I) of up to 130 mA.

The mineral composition of the initial materials and the alkali-activated binder samples was determined by X-ray diffraction analysis (XRD). The instrument used was a Bruker D8 Advance X-ray diffractometer (Bruker AXS, Karlsruhe, Germany) with Bragg–Brentano geometry, using Ni-filtered CuKα radiation and operating at up to 60 kV with a scanning step of 0.02 degrees. The Oxford Cryosystems Crystallographica Search-Match software (version 2) was used to determine the binder minerals and phase peaks. The software uses the ICDD PDF-2 database.

The mineral composition of the prime materials was analysed using the Institute of Crystallography-CNR-Bari QUALX2.0 software. The mineral phases in the software were determined using the crystallography open database (COD). Quantitative analysis using the Rietveld method was carried out with the Profex software (version 5.4.1) based on the structural data from the COD.

The microstructures of the initial materials and the alkali-activated binder samples were evaluated using scanning electron microscopy (SEM) data. The images were obtained with a Hitachi S-3400 N Type II (Hitachi, Tokyo, Japan) high-resolution SEM microscope using accelerating voltages of 5 and 15 kV.

Particle size distribution analysis was performed using a laser. The analyses were performed with the laser particle size analyser ‘Cilas 1090’ (Cilas laserandbeyond, Orléans, France) with a measuring range of 0.1–500 μm. The analysis was performed in dry dispersion mode.

Compressive strength tests of the binders were carried out using a ToniTechnik 2020.0600/132/02 (Toni Technik Baustoffprüfsysteme GmbH Berlin, Germany) computerised press. The compression test was performed according to the European standard [30]. The compression test rate was at 0.6 MPa/s for a reference area of 4 cm^2^. 

The average values of the compressive strength and the softening factor were determined from three cubes.

**Figure 4:**
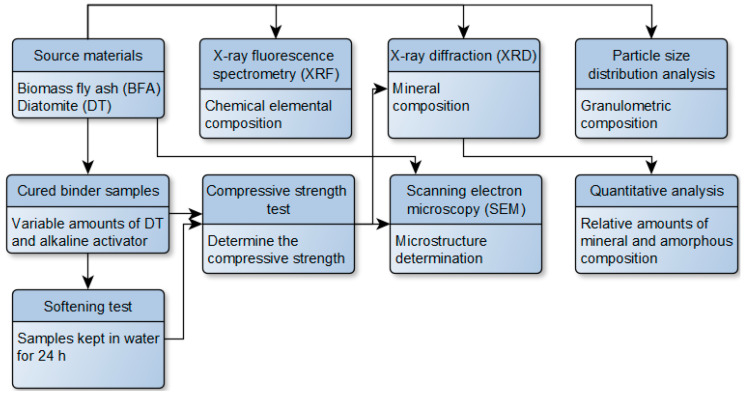
The sequence of methods and experiments applied. The arrows represent the order of the methods.

A summary of the methods/experiments used in the paper and their sequence is given in Figure 4.

## 3. Results and Discussion

### 3.1. Influence of DT Content on the Compression Strength Values

**Figure 5:**
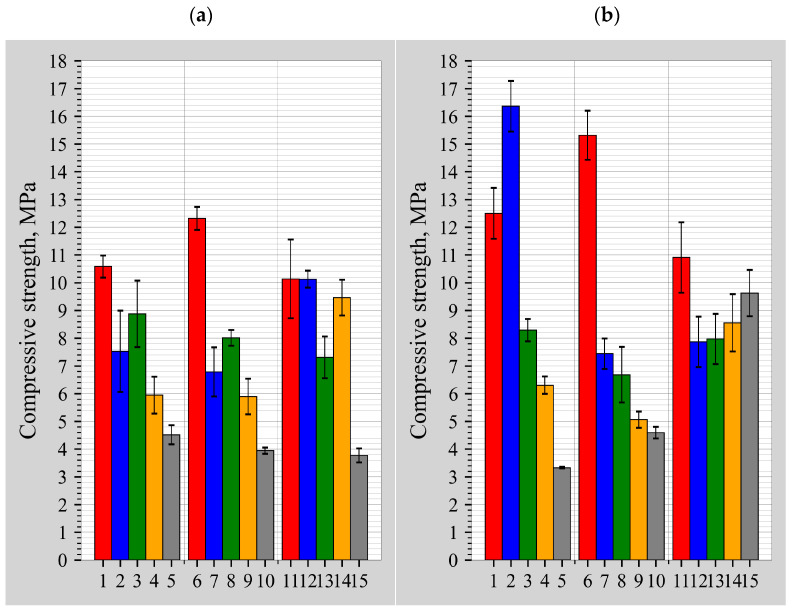
Influence of DT content on the compression strength of heated samples after 7 days (**a**) and 28 days (**b**).

It can be observed from the average compressive strength results of the samples shown in Figure 5a that the samples with the highest compressive strengths after 7 days are samples numbered 1, 6, 11, and 12. The compressive strengths of these samples ranged from 10.1 to 12.3 MPa. The comparison with other binder samples cured for 7 days shows a trend towards an increase in compressive strength independent of the amount of DT but due to the amount of alkaline activator, i.e., the highest compressive strengths were obtained with the lowest or close to the lowest amount of activator.

This trend is evident in the compressive strength results after 28 days (Figure 5b), but in this case there are more distinct differences between the groups of samples with different DT contents. The highest compressive strength values were obtained in the 10% and 30% DT content groups, that is, in samples (2 and 6) with a relatively low activator content. Samples 2 and 6 had average compressive strengths of 16.4 and 15.3 MPa after 28 days. The compressive strength after 28 days increased by more than 30% compared to the highest compressive strength value after 7 days.

**Figure 6:**
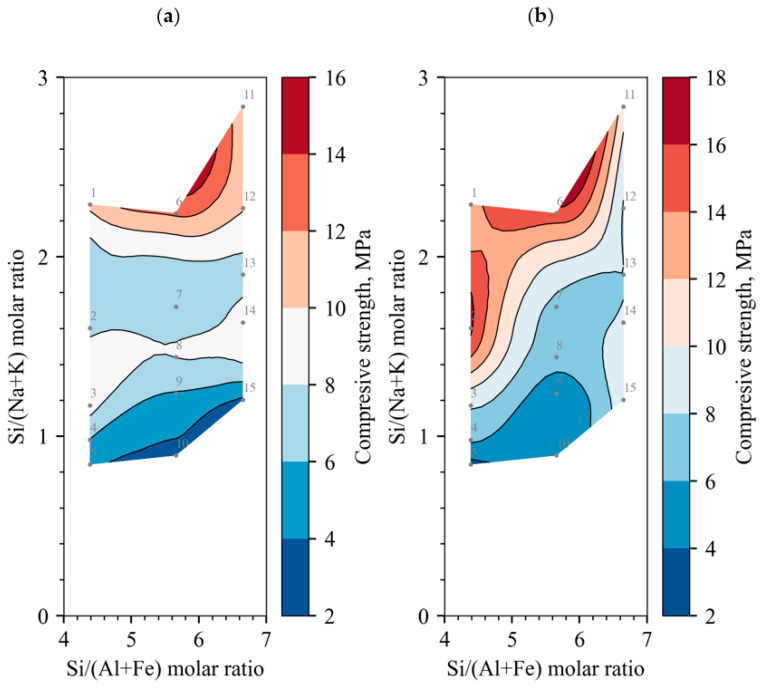
Compressive strength correlation to molar ratios without Ca cations; grey dots identify the samples number and the position: (**a**) cured for 7 days; (**b**) cured for 28 days.

**Figure 7:**
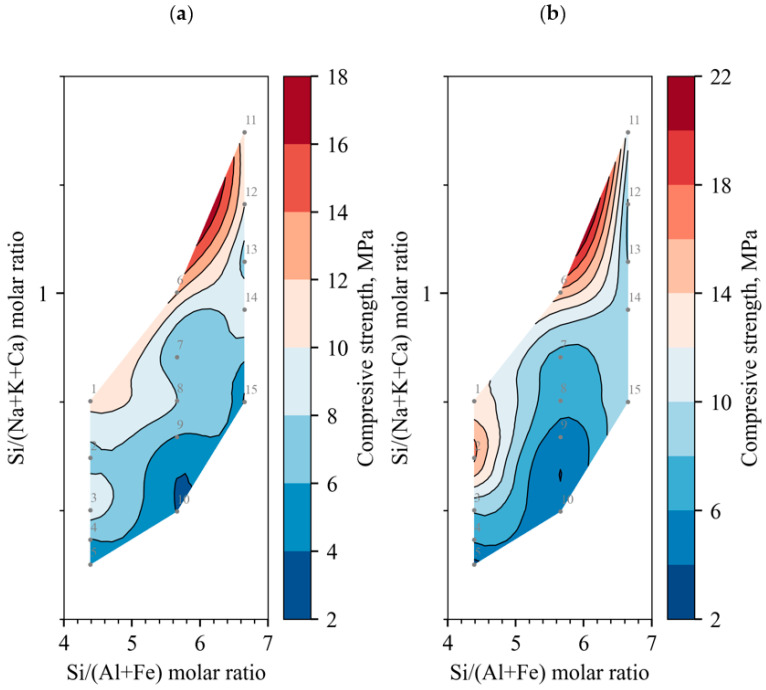
Compressive strength correlation to molar ratios with Ca cations; grey dots identify the samples number and the position: (**a**) cured for 7 days; (**b**) cured for 28 days.

Further, the dependence of the compressive strength on the molar ratios of the chemical elements is analysed: Figure 6a,b show a contour plot of the compressive strengths at 7 and 28 days, with the Si/(Al + Fe) molar ratio on the horizontal axis and the Si/(Na + K) molar ratio on the vertical axis; Figure 7a,b include the effect of Ca on the molar ratio of Si/(Na + K + Ca) on the vertical axis. Regardless of the curing time of the binder, the optimum Si/(Na + K) molar ratio of the alkali-activated concrete should be around two [31], but no increase in compressive strength was observed in Figure 6a,b. When the activating effect [32] of Ca is evaluated, the ratio decreases to 1.2, although it can be seen that there is a possible increase in compressive strength with an increasing Si/(Na + K + Ca) molar ratio, but this is not practically possible: either the Na or the Ca content must be reduced. Ca could be reduced by increasing the DT content, but the compressive results show that the compressive strength did not increase but decreased. There is no point in reducing the Na content, as the concentration is already low enough at around 5 mol/L [33]. It can therefore be concluded that BFA cannot be used as a primary binder due to its extremely high Ca content.

Another trend of increasing compressive strength can be observed in all the contours in the direction of a decreasing Si/(Al + Fe) molar ratio in the horizontal axis; according to the experience of other researchers, the optimum molar ratio should be about two [31]. To obtain such a ratio, it would be necessary to increase the content of either Al or Fe, but in this paper no source material is used that has a high content of either Al or Fe, so to improve the composition of the samples, it is necessary to use an additional source material with a dominance of Al or Fe.

The softening test reveals the effect of water on the compressive strength; i.e., if the compressive strength of the sample is reduced by more than 25%, the composition is considered to be susceptible to the effect of water. This indicates the presence of water-soluble products in the composition of such a sample or the potential for differential swelling of the bonding products, which disrupts the microstructure.

**Figure 8:**
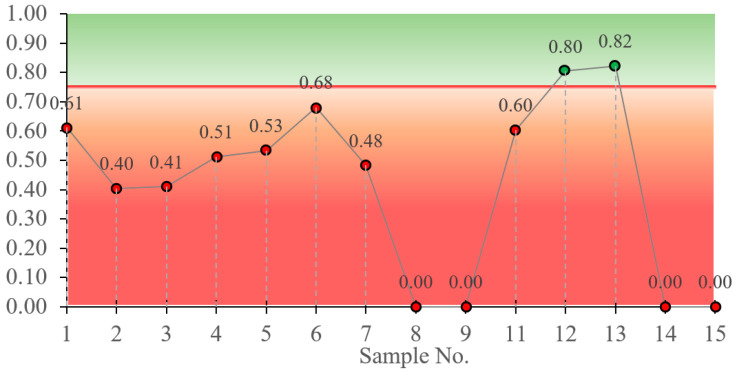
Summary of sample softening factors.

The test has highlighted several compositions (Figure 8): the samples numbered 12 and 13 are insensitive to the effects of water but, when compared with the results obtained in the compressive test, are practically useless. Although the compressive strength of test sample No. 2 was the highest obtained, the softening test shows the composition to be sensitive to water. However, test sample No. 6, which gave the best compressive results after test sample No. 2, has a softening coefficient of 0.68, which, although less than 0.75, can be considered as a potential test for more detailed investigations. The value obtained may be within the margin of error due to the small dimensions of the samples; the penetration of water may be lower in the larger samples and therefore may have less influence on the compression results, and it is necessary to carry out the analysis on a larger set of samples to reduce the relative error. From the results of the softening test, it can be summarised that the samples must be formed with a DT content greater than 10%.

### 3.2. Mineral Composition According to XRD

**Figure 9:**
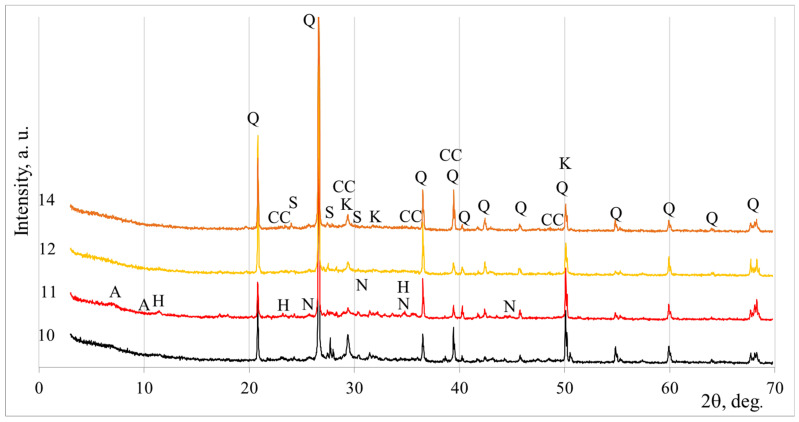
XRD patterns of alkali-activated biomass fly ash and diatomite blends. The samples numbering is from Table 4.

Four samples were selected, the maximum compressive strength of which was determined after 28 days of curing, and the mineral composition was determined by XRD analysis (Figure 9). Quartz was detected in all samples (from initial materials such as biomass ash and diatomite), which remained unreacted after alkali activation. Calcite was also detected in all samples and is likely to have formed during the curing process. When the alkali content of the system is increased (sample 2), some of the sodium hydroxide remains unreacted, and after a while it reacts with CO_2_ to form nahcolite (NaHCO_3_). Nahcolite was also detected in sample 10 because the system did not contain enough silicon compounds in the active form (only 10% by weight of DT used). The presence of nahcolite resulted in high softening values for the samples (in the geopolymer system, nahcolite was found by other researchers, such as Li et al.) [34,35]. In alkali-activated systems with diatomite, almost all the crystalline phases are derived from initial materials, as stated by Al-kroom et al. [18].

Some semicrystalline compounds form during the geopolymerisation process as well. Calcium silicate hydrate is also present as a binding component in all samples (Table 4). The presence of this mineral in the systems may explain the samples that reached the best strength values. Abdulkareem et al. [36] investigated a similar geopolymer made from biomass wood ash with a certain amount of fly ash as a substitute material. In the geopolymer, a C–S–H gel formed during the geopolymerisation process.

Additionally, in sample 2, hydrotalcite and calcium aluminium silicate hydrate, zeolite type A, were detected. These additional geopolymerisation products are closely related to the higher mechanical values of 16.4 MPa after 28 days. Zeolite phases and hydrotalcite-type phases were found in alkali-activated mixtures made from fly ash and slag, as reported by Bae et al. [37].

### 3.3. Morphology of Alkali-Activated Biomass Fly Ash and Diatomite Blends

**Figure 10:**
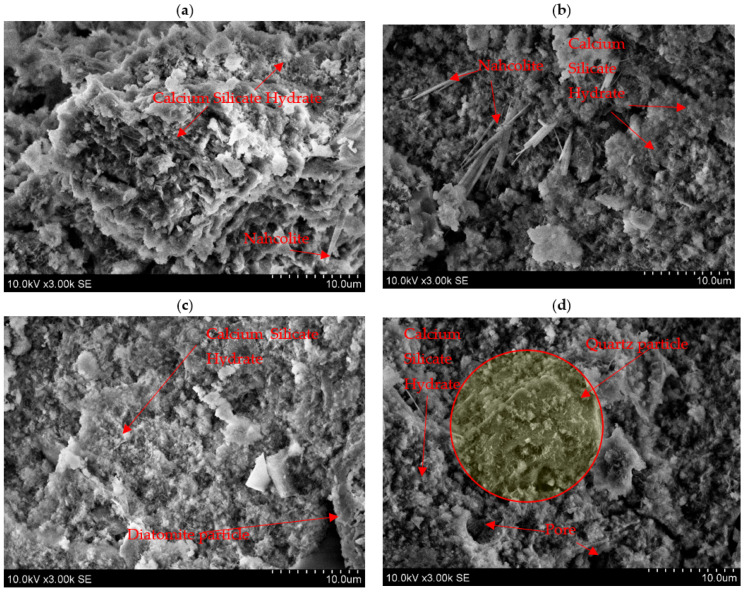
SEM images of alkali-activated BFA and DT blends. The samples numbering is from Table 4: sample 1 is (**a**); sample 2 is (**b**); sample 6 is (**c**), and sample 11 is (**d**).

Scanning electron microscope (SEM) analysis was used to evaluate the microstructure of samples after 28 days (Figure 10). The microstructure consists of heterogeneous areas composed of reacted precursors (compact matrix) and partially reacted or unreacted aluminosilicate precursors. The microstructure of calcium silicate hydrates with fibre networks, as a geopolymerisation product, dominated in all samples. Similar calcium silicate hydrate microstructures of alkali-activated fly ash geopolymers were reported by Komljenović et al. [38].

When a higher amount of sodium hydroxide was included in the systems, some of them do not react and form NaHCO_3_, which was detected as rod-like structure particles in samples 10 and 11 (Figure 10a,b). These findings are confirmed by XRD investigations. NaHCO_3_ was also found in the mineral composition (Figure 9). In the study [39], the microstructure of NaHCO_3_ particles has a rod-like nature.

In the samples with higher amounts of DT (30 and 50 wt%), unreacted phases are also visible, and these phases could be assigned to quartz and diatomite particles (Figure 10c,d). Thus, in the samples studied, diatomite and quartz were identified as unreacted phases, while C-S-H and nahcolite were identified as geopolymerisation products. Similar findings were reported by Topçu et al. [40], who found that after the geopolymerisation process, unreacted particles from coal bottom ash precursors were detected. The additions of DT (10 and 30 wt%) improved the microstructure of alkali-activated biomass fly ash; a denser microstructure improves the compression strength of the samples. As Ilkentapar et al. [21] also found, the results were similar.

The principal findings of analogous studies are summarised in Table 5. Samples of alkali-activated biomass fly ash cured at ambient temperature exhibited low compressive strength, with values ranging from 3.50 to 3.50 MPa after 28 days [1,6]. As demonstrated in the study [4], it is possible to achieve 51.00 MPa for samples cured at ambient temperature by utilising more reactive biomass ash, such as rice husk and bark ash. It was established that slightly higher strength values (51.00 MPa) were associated with curing at higher temperatures.

**Table 5:** The influence of the composition of the initial materials and the curing conditions on the strength characteristics of hardened samples.

Precursor	Alkaline Activator	Curing Conditions	Maximal Compressive Strength, MPa	Reference
Biomass fly ash from a biomass power plant	Na_2_CO_3_ and Na_2_SiO_3_	ambient temperature for 28 days	3.08	[1]
High-carbon biomass ash	Na_2_CO_3_ and Na_2_SiO_3_	ambient temperature for 28 days	3.50	[6]
Rice husk and bark ash	NaOH and Na_2_SiO_3_ solution	ambient temperature for 28 days; ambient temperature for 27 days with 60 °C for 24 h	51.00; 56.00	[4]
Metakaolin and biomass wood ash	NaOH and Na_2_SiO_3_ solution	ambient temperature for 28 days	83.00	[9]
Coal fly ash and biomass wood ash	NaOH and Na_2_SiO_3_ solution	ambient temperature for 28 days	50.38 (mortar)	[11]
Glass powder and wood biomass ash.	NaOH solution	ambient temperature for 28 days	19.62 (after 14 days)	[12]
Coal fly ash and diatomite	NaOH solution	heat curing at 75 °C for 24 h	38.40 (mortar)	[18]
Calcined diatomite and biomass wood fly ash.	NaOH solution	20 °C for 27 days with 60 °C for 24 h	16.40	This study

In order to enhance the mechanical properties, it is recommended that the utilisation of a combination of precursors be employed in the process of extracting the aluminium silicate material [9,11,12,18]. In this study, biomass wood fly ash and calcined diatomite were utilised as precursors. The mixture was found to be alkali-active in a NaOH solution, and the maximum compressive strength was achieved, with a value of 16.40 MPa. It is conceivable that in future studies, the triple precursor could be utilised by incorporating aluminosilicate initial materials in their active form.

## 4. Conclusions

- The maximum average compressive strength after 7 days ranged from 10.1 to 12.3 MPa, and after 28 days, it ranged between 15.3 and 16.4 MPa. The highest compressive strength was obtained at the lowest or near-lowest activator level (5 mol/L), irrespective of the DT content for the samples cured for 7 days. The low amount of alkalinity required can be explained by the fact that the Ca^2+^ cations present in BFA act as an activator. The strength of the binders cured for 28 days was dependent on the amount of DT: the best results were obtained at DT contents of 10 and 30%.
- From the analysis of the dependence of the compressive strength on the molar ratios of chemical elements, it was observed that the maximum average compressive strength was not obtained at the optimal Si/(Na + K) molar ratio of the alkali-activated binder, which was ~2. When Ca moles are evaluated, the ratio decreases to 1.2; in order to achieve the optimal ratio, it is necessary to reduce the amount of either Na or Ca. This can be achieved only by changing the main binder component from BFA to DT. Another tendency for the compressive strength to increase is observed when the Si/(Al + Fe) molar ratio decreases. In order to achieve the optimal composition ratio (~2), it is necessary to increase the amount of Al and Fe; therefore, it is necessary to use an additional raw material in the composition in which Al or Fe predominates.
- The performed softening test also repeatedly confirms that the main binder component should be changed from BFA to DT, since samples containing less than 30% DT are unsuitable. Even for samples containing 30% DT, only one composition had a result at the borderline, within the error range.
- XRD analysis showed quartz and calcite from aluminosilicate precursors such as DT and BFA. Both of these compounds are non-reactive, so samples with a higher alkali content contain a certain amount of nahcolite. Despite the non-reactive compounds, calcium silicate hydrate, hydrotalcite, and calcium aluminium silicate hydrate (zeolite A type) formed after geopolymerisation. These additional geopolymerisation products are closely related to higher mechanical properties.
- SEM analysis confirmed the XRD results and showed that DT additives (10 and 30 wt%) improved the microstructure of alkali-activated BFA, which is closely related to compressive strength values.
- Although the practical application of the alkali-activated concrete produced from BFA and DT in this work is rather limited, such concrete could be used for artificial aggregates or small architectural elements.

Future research in this study could focus on improving the composition of the aluminosilicate precursor by including initial materials dominated by silicon and aluminium in their active form. Other researchers could concentrate on other properties of alkaline-activated concrete, such as long-term properties (shrinkage and creep), workability, and durability.

## Figures and Tables

**Table 1:** Chemical composition of initial materials for the precursor, according to XRF analysis (wt%).

	SiO_2_	CaO	K_2_O	Al_2_O_3_	MgO	SO_3_	P_2_O_5_	Fe_2_O_3_	Cl	Na_2_O	MnO	TiO_2_	BaO	ZnO	SrO	ZrO_2_	Other/LOI
**BFA**	32.99	32.63	7.48	5.91	5.57	5.38	4.26	2.87	0.681	0.672	0.613	0.495	0.185	0.126	0.044	0.042	0.052/0.042
**DT**	84.5	0.37	1.83	7.69	1.43	0.03	-	3.16	-	0.34	-	-	-	-	-	-	0.65

**Table 2:** Mineral composition of initial materials for the precursor, according to Rietveld analysis (wt%).

Symbol	COD Number	Mineral Name	Formula	Quantity, %	Crystalline Phase, %
BFA					
Q	901–0146	Quartz	SiO_2_	77.64	78
C	901–6705	Calcite	CaCO_3_	5.97	
B	901–5087	Cristobalite	SiO_2_	8.16	
L	101–1094	Lime	CaO	3.19	
M	900–0701	Microcline	KAlSi_3_O_8_	1.55	
P	900–0492	Periclase	MgO	1.10	
A	412–4074	α-quartz	SiO_2_	2.39	
Amorphous (between 20 and 43 deg)					22
**DT**					
Q	901–0146	Quartz	SiO_2_	91.80	69.8
U	901–2886	Muscovite	Ca_0.011_K_0.776_ Na_0.181_Al_2.726_Fe_0.03_ Mg_0.02_Si_3.15_Ti_0.02_O_11_	6.00	
S	153–5821	Low sanidine ferroalumosilicate	Fe_0.28_KAl_0.72_Si_3_O_8_	2.20	
Amorphous (between 14.8 and 36 deg)					30.2

**Table 3:** Mix designs of initial materials.

Sample No.	BFA Weight, %	DT Weight, %	Weight Ratio NaOH/(BFA + DT)	NaOH Solution Concentration, mol/L	Weight Ratio Total Water/(BFA + DT)
1	100	10	0.05	3.4	0.35
2			0.09	6.3	0.35
3			0.14	10.8	0.32
4			0.17	13.7	0.32
5			0.21	16.5	0.31
6	100	30	0.08	5.5	0.37
7			0.12	8.3	0.35
8			0.15	10.5	0.35
9			0.18	12.6	0.35
10			0.26	17.9	0.37
11	100	50	0.07	4.4	0.42
12			0.10	6.5	0.39
13			0.13	8.2	0.39
14			0.15	9.9	0.38
15			0.22	14.2	0.39

**Table 4:** Mineral composition of selected samples, according to XRF.

Symbol	PDF-2 Number	Mineral Name	Formula
**Sample No. 1**			
Q	85–797	Quartz	SiO_2_
CC	81–2027	Calcite	CaCO_3_
K	33–306	Calcium Silicate Hydrate	Ca_1.5_SiO_3.5_∙xH_2_O
N	1–909	Nahcolite	NaHCO_3_
**Sample No. 2**			
Q	85–797	Quartz	SiO_2_
K	33–306	Calcium Silicate Hydrate	Ca_1.5_SiO_3.5_∙xH_2_O
CC	81–2027	Calcite	CaCO_3_
N	1–909	Nahcolite	NaHCO_3_
A	76–1507	Calcium aluminium silicate hydrate (zeolite A)	Ca_5.57_ Al_12.3_Si_12_O_49.2_ H_2.34_
H	22–700	Hydrotalcite	Mg_6_Al_2_CO_3_(OH)_16_∙4H_2_O
**Sample No. 6**			
Q	85–797	Quartz	SiO_2_
K	33–306	Calcium Silicate Hydrate	Ca_1.5_SiO_3.5_∙xH_2_O
CC	81–2027	Calcite	CaCO_3_
**Sample No. 11**			
Q	85–797	Quartz	SiO_2_
K	33–306	Calcium Silicate Hydrate	Ca_1.5_SiO_3.5_∙xH_2_O
CC	81–2027	Calcite	CaCO_3_

## Data Availability

The original contributions presented in this study are included in the article. Further inquiries can be directed to the corresponding author.
